# Seroprevalence of Canine Ehrlichiosis and Microscopic Screening for Canine Babesiosis in Dogs in Harare, Zimbabwe, 2016-2017

**DOI:** 10.1155/2019/4130210

**Published:** 2019-12-01

**Authors:** Solomon Dhliwayo, Brighton Chihambakwe, Knowledge Taonezvi, Silvester M. Chikerema, Musavengana T. Tivapasi, Davies M. Pfukenyi

**Affiliations:** Department of Clinical Veterinary Studies, Faculty of Veterinary Science, University of Zimbabwe, P.O. Box MP 167, Mount Pleasant, Harare, Zimbabwe

## Abstract

A cross-sectional study was done to determine ehrlichiosis seroprevalence and babesiosis prevalence in dogs that were presented to selected veterinary clinics in Harare. Sera from randomly selected dogs were tested for antibodies to *Ehrlichia* spp. using an enzyme-linked immunosorbent assay while microscopy of peripheral blood smears was used to confirm babesiosis. Overall, 75.2% (88/117, 95% CI: 66.2–82.5) of sera samples tested were positive to *Ehrlichia* spp. antibodies while the prevalence of canine babesiosis was 47.9% (56/117, 95% CI: 38.6–57.3). Age, breed, and sex were found not to be associated with the two disease conditions (*p* > 0.05). Most of the dogs with babesiosis (82.1%, 46/56) were also positive to *Ehrlichia* spp. antibodies. Hypoalbuminaemia (53.8%, 63/117), anaemia (53.0%, 62/117) and thrombocytopaenia (40.2%, 47/117) were the most common laboratory findings. Thrombocytopaenia and hypoalbuminaemia was more pronounced in dogs with babesiosis only while anaemia was more marked in dogs with babesiosis and positive to *Ehrlichia* spp. antibodies.

## 1. Introduction

Canine ehrlichiosis, a potentially fatal disease of dogs is caused by *Ehrlichia* species. The disease has acute, subclinical and chronic stages [1] and clinical findings in dogs vary with the stage of the infection [2]. Clinical signs observed in the acute phase of the disease include fever, anorexia, oculo-nasal discharges, vomiting, weight loss, hepatosplenomegaly, lymphadenopathy and, rarely epistaxis and haemorrhage [1, 3]. The chronic phase is marked by epistaxis, haematuria, petechiae, ecchymosis distributed over skin surface, respiratory distress, ocular abnormalities, and CNS signs [1]. Previous canine ehrlichiosis studies in Zimbabwe showed an overall seroprevalence of 42% [4]. Dogs are naturally infected with several species; *E. canis*, *E. chaffeensis*, and *E. ewingii* [5] with *E. canis* being the most common and causing the most severe clinical disease in Africa and Asia [6]. Although several *Ehrlichia* spp. are able to cause natural disease in dogs, only *E. canis* and *E. ruminantium* are known to occur in southern Africa [6]. However, serological evidence of antibodies against *E. chaffeensis*, *E. ewingii*, and *E. ruminantium* from dog sera in South Africa and Zimbabwe has been documented [7–10]. Some studies from Venezuela and Costa Rica have suggested that *E. canis* might be zoonotic [11, 12].

Canine babesiosis is a disease of worldwide significance that causes fever, haemolytic anaemia, haemoglobinuria and death [13]. It is an important disease of domestic and wild canidae [14]. The most common clinical signs associated with babesiosis are anorexia, fever, depression/lethargy, pale mucosae, splenomegaly, and weight loss [13]. Canine babesiosis studies in Zimbabwe are limited, with two studies reporting a prevalence of 6.9% and 26% [4, 15]. The disease is caused by three strains of the large *Babesia canis* namely, *B. canis*, *B. rossi*, and *B. vogeli*; the small *B. gibsoni* and the micro*babesia*e; *B. microti*, *B. vulpes* and *B. conradae* [16]. In Africa, the small-sized *Babesia* has been reported in East and North Africa [13, 17, 18] with the rest of Africa reporting the large-sized *Babesia* [13, 19, 20] and there is no report of the micro*babesiae* [21, 22]. However, currently there is no literature on *Babesia* spp. infecting dogs in Zimbabwe.

Dogs can have concurrent infections with various *Babesia*, *Bartonella*, *Ehrlichia* and *Rickettsia* species [23] and those with a heavy tick exposure can be infected at a higher rate with multiple and potentially zoonotic tick-borne pathogens [23]. Worldwide, tick-borne diseases are an important cause of morbidity and mortality in dogs with the brown dog tick, *Rhipicephalus sanguineus* being implicated as a vector of *E. canis*, *B. vogeli*, and *B. gibsoni* [1, 22]. Hence, the transmission occurs when *R. sanguineus* takes a blood meal from the dog [1, 2]. Concurrent infections of *E. canis* with *Babesia* spp. have been reported [4, 24, 25] leading to more severe case outcomes [26]. The epidemiology of canine tick-borne diseases may change due to the effects of climate change and the ease of international travel [27]. Studies about the prevalence of *Babesia* and *Ehrlichia* spp. co-infections in dogs in Zimbabwe are limited [4]. The first objective of this study was to determine the seroprevalence of ehrlichiosis and the prevalence of babesiosis. The second objective was to determine the prevalence of *Ehrlichia* spp. seropositivity in dogs with babesiosis and the common clinicopathological findings.

## 2. Materials and Methods

### 2.1. Study Location, Design and Collection of Blood Samples

This study was conducted in urban Harare, Zimbabwe where a cross-sectional study was employed to collect blood samples from dogs between October 2016 and March 2017. The blood samples were collected from dogs presented for routine elective surgery or ill-health at randomly selected private veterinary practices. A systematic random sampling technique was used to select dogs presented at the selected private veterinary practices; the first dog being selected using simple random sampling and every tenth dog thereafter. The selected dogs were restrained manually and whole blood was collected from the cephalic vein into 5 ml plain and ethylene diamine tetra-acetic acid (EDTA) tubes. Serum obtained through centrifugation at 2500 rpm for 10 minutes using a Sigma 3E-1 centrifuge (Sigma Harz, Germany) was stored at −20°C prior to use for *Ehrlichia* spp. serological testing. The EDTA blood was used for complete blood counts and microscopy of peripheral blood from an ear vein was used for detection of *Babesia* piroplasms from Giemsa-stained thin blood smears. Data recorded during blood samples collection included age, sex, and breed of the dog.

### 2.2. Testing for Ehrlichia spp. Antibodies and Babesia Piroplasms

The ImmunoComb® Canine *Ehrlichia* Antibody Test Kit (Biogal-Galed Laboratories, Israel) was used to detect IgG antibodies against *Ehrlichia* spp. from the collected dog sera. The test was carried out according to the manufacturer's instructions (http://www.biogal.co.il) and as previously described [28, 29]. The results were read with a calibrated colour Comb Scale (graded S0–S6), which was provided with the test kit. A scale of S3, which is equivalent to a positive immune response at a titre of 1 : 80 by an indirect fluorescent antibody (IFA) test, was considered as the “cut-off” level of IgG antibodies (http://www.biogal.co.il). Hence, in this study serum samples with a Comb Scale score of ≥S3 (i.e. ≥1 : 80 titre) were considered to be positive for *Ehrlichia* spp. antibodies.

Microscopy was used for the detection of *Babesia* piroplasms. Giemsa-stained, thin peripheral blood smears were prepared from peripheral blood from an ear vein. Two peripheral blood smears were made and from each, a minimum of 100 fields was microscopically examined for the presence of *Babesia* piroplasms. Careful examination of the Giemsa-stained thin peripheral blood smears was done by well-trained and experienced technologists.

### 2.3. Haematology and Clinical Chemistry

EDTA blood of dogs testing positive for *Babesia* and *Ehrlichia* spp. antibodies was subjected to a complete blood count using an automated haematology analyzer (BC-2008 Vet-Shenzhen Mindray Bio-medical Electronics, China). The blood samples were analyzed to measure haematocrit (HCT), total number of erythrocytes (RBC), haemoglobin concentration (HB), mean erythrocyte volume (MCV), mean corpuscular haemoglobin content (MCH), mean haemoglobin concentration in erythrocytes (MCHC), erythrocyte distribution width (RDW), and total number of platelets (PLT). The leukogram measurement included total number of leukocytes (WBC), absolute number of neutrophils (NEU), lymphocytes (LYM), monocytes (MONO), and eosinophils (EOS). An automated chemistry analyzer (Humastar 180®—Human GmbH, Wiesbaden, Germany) was used to measure albumin and globulin. Reference values for all the parameters were adopted from Diagnopath Veterinary laboratory (Pvt) Ltd, Harare.

### 2.4. Data Analysis

The recording and editing of the data was performed using Microsoft Excel®. A statistical package called Epicalc (2000) version 2, was used to analyze all the raw data. The total number of positive/seropositive animals was calculated from the total number of samples tested over the study period and expressed as a percentage. Positive/seropositive animals were examined in relation to sex, breed, and age. The Chi-square test was used to measure differences in proportions between generated categories of babesiosis and ehrlichiosis status and values of *p* < 0.05 were considered as significant. Seropositivity/positivity was also analyzed according to HCT, RBCs, MCV, hemoglobin, MCHC, platelets, WBCs, total protein, and albumin. Association between the above blood parameters was evaluated by calculating the Chi-square test, relative risk and the 95% confidence interval using Epicalc version 2.

## 3. Ethical Considerations

Ethical approval for use of dogs and for all protocols in this study was obtained from the Ethical and the Higher Degree committees of the Faculty of Veterinary Science reference number VEHDC 2016/05. The purpose of this study was well explained to the veterinary practitioners stationed at all the veterinary clinics in Harare, who all expressed consent to participate in the study. Verbal and written consent was obtained from owners whose dogs were selected for the study. Standard operating procedures were followed for collection of blood samples.

## 4. Results

### 4.1. Canine Ehrlichiosis Seroprevalence and Babesiosis Prevalence

Tables 1 and 2 show the distribution of sampled dogs, ehrlichiosis seroprevalence and babesiosis prevalence according to different categories. A total of 117 serum samples were collected and the overall ehrlichiosis seroprevalence was 75.2% (88/117; 95% CI: 66.2–82.5) whilst babesiosis prevalence was 47.9% (56/117; 95% CI: 38.6–57.3). Seropositivity/positivity to the two diseases was found not to be associated (*p* > 0.05) with the age, breed, sex and health status of the dogs. Of the ehrlichiosis seropositive dogs, 65.9% (58/88) had a titer of 1 : 80 and 34.1% (30/88) a titer of >1 : 80. Five and seven dogs that were ehrlichiosis seropositive had a titer of 1 : 160 and 1 : 320, respectively. Of the total serum samples tested, 39.3% (46/117; 95% CI: 30.5–48.8) were *Babesia* spp. positive and also seropositive to *Ehrlichia* spp. Most of the dogs with babesiosis (82.1%, 46/56) were positive to *Ehrlichia* spp. antibodies.

### 4.2. Haematological Findings

There were no significant differences (*p* > 0.05) noted on the erythrogram and leukogram of apparently healthy and ill dogs. The changes of the erythrogram and leukogram are presented in Table 3. Hypoalbuminaemia (53.8 %, 63/117), anaemia (53.0%, 62/117), and thrombocytopaenia (40.2%, 47/117) were the most common laboratory findings. Anaemia, evidenced by decreased HCT, absolute RBC and HB values was the main erythrogram change noted. A significantly (*p* < 0.01) higher percentage of dogs positive to both *Babesia* and *Ehrlichia* spp. recorded decreased values of these parameters compared to those seropositive to *Ehrlichia* spp. only. The anaemia, as measured by the decreased mean HCT was more pronounced in those dogs positive to both *Babesia* and *Ehrlichia* spp. (24.1 ± 0.6) compared to those positive to *Babesia* spp. only (25.3 ± 1.4) and *Ehrlichia* spp. only (27.6 ± 2.8). On the leukogram, eosinopaenia and monocytopaenia, accompanied with a neutrophilic and lymphocytic leukocytosis were the main changes noted. Lymphocytosis and neutrophilia was more pronounced in dogs positive to both *Babesia* and *Ehrlichia* spp. However, no significant differences (*p* > 0.05) in the values of these parameters were noted among the different groups of dogs.

Thrombocytopaenia, as measured by decreased platelets count was recorded in 52% of dogs positive to both *Babesia* and *Ehrlichia* spp., 30% and 26% of those positive to *Babesia* spp. only and *Ehrlichia* spp. antibodies only, respectively. The difference in percentages was significant (*p* < 0.05) between dogs positive to both *Babesia* and *Ehrlichia* spp. and those positive to *Ehrlichia* spp. antibodies only. The decreased mean platelets count was lowest for those positive for *Babesia* spp. only (116.7 ± 21.5), followed by dogs positive to both *Babesia* and *Ehrlichia* spp. (134 ± 18.7) and lastly for those positive to *Ehrlichia* spp. antibodies only (139 ± 22.3) but the differences were not significant (*p* > 0.05).

Eighty percent of dogs with babesiosis only, 69.6% positive to both *Babesia* and *Ehrlichia* spp. and 45.2% with ehrlichiosis only had hypoalbuminaemia as measured by a decreased value of albumin. The difference in percentages was significant (*p* < 0.05) between the dogs positive to both *Babesia* and *Ehrlichia* spp. and those positive for *Ehrlichia* spp. antibodies only. The hypoalbuminaemia was more pronounced in those positive to *Babesia* only (21.9 ± 1.5) compared to dogs positive to both *Babesia* and *Ehrlichia* spp. (23.8 ± 0.7) and *Ehrlichia* spp. antibodies only (24.2 ± 0.8).

## 5. Discussion

The lack of significant differences noted on the erythrogram and leukogram of apparently health and ill dogs was an unexpected finding. This was probably because the ill dogs had a low grade of babesiosis and/or ehrlichiosis infection. Due to lack of clinical details and follow-up of ill dogs, the intensity of babesiosis and/or ehrlichiosis infection could not be determined. In addition, the *Babesia* spp. parasite load was not determined and hence, an inability to assess the intensity of infection. *Babesia vogeli* causes a mild to moderate disease associated with mild laboratory changes [13]; it is possible that the ill dogs were infected by *B. vogeli* but the current study did not determine the *Babesia* spp. in babesiosis positive dogs. In naturally infected dogs, mild haematological abnormalities occur in the subclinical phase of ehrlichiosis [6, 30]; it is therefore likely that apparently health dogs had a subclinical infection whilst the ill ones had a low grade ehrlichiosis infection. It is also important to note that ehrlichiosis serology is indicative of exposure and not necessarily acute infection. Since no definitive diagnosis was determined, the presence of other diseases could have masked the observed erythrogram and leukogram findings. Hence, the findings of this study should be viewed in the light of its limitations.

The most frequently used diagnostic technique for *Ehrlichia* spp. infection confirmation in the dog is serology [2]. Kahn et al. [31] showed that the indirect fluorescent antibody (IFA) and ELISA tests were equally sensitive for the early detection of IgG antibodies against *E. canis*. The ImmunoComb® Canine *Ehrlichia* Antibody Test kit has a very high sensitivity (100%) and a high specificity (94.1%) (http://www.biogal.co.il) and hence, reduces the possibility of false positive and false negative reactions. Based on the above observations and its use in earlier studies [2, 29], the ImmunoComb® Canine *Ehrlichia* Antibody Test kit was therefore used to determine IgG antibodies to *Ehrlichia* spp. in dogs in the study area. However, the use of serology was a limitation of the study; it is indicative of exposure and not necessarily acute infection, hence the stage of infection could not be determined. In addition, ehrlichiosis seroprevalence could probably have been over-estimated as dogs with *Ehrlichia* infection will self-cure and remain seropositive for variable periods thereafter [6]. Cross-reactivity between antibodies against *Ehrlichia* spp. has been reported [7–9] and in areas where they co-exist their serological differentiation may therefore not be possible and this is a limitation to our study. The use of molecular techniques (PCR and sequencing) using parasite-specific primers would have provided a better diagnostic tool in terms of both sensitivity and specificity and along with the identification of the infecting species.

Blood smear examination is a useful diagnostic tool for clinical babesiosis in dogs and microscopy evaluation continues to be the easiest and most accessible diagnostic test for most laboratories [32]. Although microscopy is highly specific and can be used to diagnose the large forms of *Babesia*, the small piroplasms are hard to observe by light microscopy which has poor to moderate sensitivity and expertise is needed [22, 33]. In addition, the limit of parasites detection in a thin blood smear is reported to be parasitemias of 0.5% [34]. For these reasons, our study might have underestimated the prevalence of babesiosis in the studied dogs and more sensitive molecular PCR-based methods should be used in future studies.

Our study found a higher ehrlichiosis seroprevalence (75.2%) than that previously (52%) recorded [4]. Similarly, the current study reported a higher canine *Babesia* prevalence (47.9%) than an earlier report of 26% [4]. A recent PCR-based study on *Babesia* in apparently healthy rural dogs in the country also found a low prevalence (6.9%) of *Babesia* spp. antigen [15]. The prevalence was also much higher than that reported by Rautenbach et al. [35]. Differences in study areas and in testing regimes (e.g. for *Ehrlichia* spp.) could probably account for the variations. *Babesia* spp. identification was a limitation of the present study and there is no literature on the species occurring in the country. However, *B. gibsoni* and *B. rossi* have been confirmed in neighbouring Zambia [14] while *B. rossi* and *B. vogeli* are known to occur in South Africa [13]. Further studies are required to determine the tick vectors and, the *Babesia* and *Ehrlichia* spp. present in dogs in the country and also to assess their roles in the clinical and pathological picture of the diseases. In support of earlier observations in the country [4], co-presence was shown with over 80% of *Babesia* spp. positive dogs also being seropositive to *Ehrlichia* spp. antibodies. The ehrlichiosis/babesiosis concurrent prevalence found in this study (39.3%) was more than twice that was previously reported (17%) in the country [4] and this phenomenon has been reported elsewhere [25, 36–38]. *Rhipicephalus sanguineus*, the brown dog tick transmits both parasites [37] and this is likely to account for the observed findings.

Anaemia and thrombocytopaenia have been demonstrated as consistent laboratory findings in canine babesiosis and ehrlichiosis [13, 25, 36, 39, 40]. In an earlier study in the country, the two were the most common laboratory abnormalities observed in dogs with *Babesia* and *Ehrlichia* infection [4]. Despite having no signs of the disease, *Babesia* and *Ehrlichia* positive dogs in the current study had anaemia and thrombocytopaenia. However, thrombocytopaenia was observed in less than a third of dogs with either babesiosis or ehrlichiosis but in more than half of those with both. In Zambia, thrombocytopaenia was also observed in less than one-fifth of *Babesia* infected dogs [14]. The low number of dogs with thrombocytopaenia might be probably due to the fact that serology was performed for ehrlichiosis which indicates past exposure rather than active infection.

In this study, leukocyte abnormalities were nonspecific and most dogs had eosinopaenia and monocytopaenia while neutrophilia, lymphocytosis, and leukocytosis were observed in some of the dogs. Niwetpathomwat et al. [36, 40] also observed nonspecific leukocyte abnormalities in *Babesia* and *Ehrlichia* infected dogs. The observed abnormalities of eosinopaenia, monocytopaenia and neutrophilia are in agreement with other studies for ehrlichiosis [37, 39]. One of the predominant biochemical abnormalities found in dogs infected with *E. canis* is hypoalbuminaemia [38, 41]. In this study, hypoalbuminaemia was constantly observed in dogs either positive to one or both pathogens.

Thrombocytopaenia and hypoalbuminaemia was more pronounced in dogs positive to *Babesia* spp. only while anaemia was more marked in those positive to both *Babesia* and *Ehrlichia* spp. The severity of anaemia and thrombocytopaenia in *Babesia*–*Ehrlichia* concurrent infections was intermediate to that of individual infections [4]. In *Babesia*–*Ehrlichia* mixed infection the disease was reported to be more severe [42]. Manzillo et al. [43] reported that anaemia and thrombocytopaenia are more common in dogs with babesiosis than those with ehrlichiosis. Single infections with *B. gibsoni* or *B. canis* gave more severe haematological results than mixed infections [14]. The observed differences in severity of the laboratory abnormalities are likely dependent on *Babesia*/*Ehrlichia* spp. in individual and concurrent infections. Hence, it is difficult to ascribe haematological abnormalities to any of the *Babesia*/*Ehrlichia* spp. Tsachev et al. [37] indicated that variations in haematological profiles in *Ehrlichia* infected dogs may be related to differences in the virulence of *Ehrlichia* spp. strains, antigen heterogeneity of this bacterial agent and the clinical form of the disease. In this study, no particular stage of ehrlichiosis was selected for evaluation.

The laboratory abnormalities observed, particularly anaemia and thrombocytopaenia can affect the outcome of routine surgeries [30]. Hence, before such procedures are done, testing for *Ehrlichia*/*Babesia* in dogs from the study area should be considered. According to Kelly [6], although apparently healthy, dogs subclinically infected with *E. canis* usually have laboratory abnormalities, especially thrombocytopaenia, anaemia, hypoalbuminaemia and leukocytosis singly or in combination. In our study, the dogs positive to *Ehrlichia* spp. antibodies were apparently healthy and had the above mentioned laboratory abnormalities further supporting that many natural *E. canis* infections are subclinical [26].

In conclusion, this study showed a high prevalence of *Ehrlichia*/*Babesia* presence in the study dogs. The haematology and biochemical profiles are similar to the results observed in other reports with the most significant abnormalities being anaemia, thrombocytopaenia and hypoalbuminaemia. Thrombocytopaenia and hypoalbuminaemia was more pronounced in dogs with *Babesia* only while anaemia was more marked in those positive to both *Babesia* and *Ehrlichia* spp. However, there were no laboratory abnormalities which could be utilized to differentiate between individual and concurrent presence. Further studies are required to determine the *Babesia* and *Ehrlichia* spp. present in dogs in the country.

## Figures and Tables

**Table 1:** Distribution of ehrlichiosis seroprevalence according to age, breed, and sex.

Category	Level	Number tested	Positive	Seroprevalence (%)	95% cnfidence interval
All animals	Overall	117	88	75.2	66.2–82.5

Age	Puppy	27	17	63.0^a^	42.3–79.9
	Adult	83	66	79.5^a^	69.0–87.3
	Geriatrics	7	5	71.4^a^	30.3–94.9

Breed	Small	12	7	58.3^a^	28.6–83.5
	Large	73	59	80.8^a^	69.6–88.6
	Mixed	26	22	84.6^a^	64.3–95.0

Sex	Female	56	42	75.0^a^	61.4–85.2
	Male	61	46	75.4^a^	62.4–85.2

Health status	Apparently health	70	51	72.9^a^	60.7–82.5
	Ill	47	37	78.7^a^	63.9–88.8

Figures with the same superscript for each category are not significantly different at *p* < 0.05.

**Table 2:** Prevalence of babesiosis according to age, breed, and sex.

Category	Level	Number tested	Positive	Prevalence (%)	95% confidence interval
All animals	Overall	117	56	47.9	38.6–57.3

Age	Puppy	27	13	48.1^a^	29.2–67.7
	Adult	83	40	48.2^a^	37.2–59.4
	Geriatrics	7	3	42.9^a^	11.8–79.8

Breed	Small	12	7	58.3^a^	28.6–83.5
	Large	73	35	47.9^a^	36.2–59.9
	Mixed	26	14	53.8^a^	33.8–72.9

Sex	Female	56	23	41.1^a^	28.4–55.0
	Male	61	33	54.1^a^	40.9–66.7

Health status	Apparently health	70	33	47.1^a^	35.2–59.4
	Ill	47	23	48.9^a^	34.3–63.7

Figures with the same superscript for each category are not significantly different at *p* < 0.05.

**Table 3:** Percent of dogs with below and above the normal range measured haematological parameters according to infection status.

Parameter	Normal range	% dogs with values below [above] normal range		
		Ehrlichiosis only (*n* = 42)	Babesiosis only (*n* = 10)	Co-infection (*n* = 46)
HCT (%)	37–55	35.7 [9.5]	70.0 [0.0]	82.6 [2.2]
RBC (10^12^/L)	5.5–8.5	33.3 [0.0]	50.0 [0.0]	71.7 [0.0]
HB (g/L)	12–18	33.3 [19.0]	70.0 [0.0]	67.4 [2.2]
MCV (fL)	60–77	7.1 [2.4]	0.0 [30.0]	4.3 [13.0]
MCH (pg)	19–23	0.0 [61.9]	10.0 [30.0]	4.3 [58.7]
MCHC (g/L)	32–36	7.1 [19.0]	40.0 [0.0]	15.2 [19.6]
RDW (%)	11.6–14.8	14.3 [35.7]	40.0 [20.0]	17.4 [32.6]
PLT (10^9^/L)	200–500	26.2 [2.4]	30.0 [0.0]	52.2 [0.0]

WBC (10^9^/L)	6–17	2.4 [26.2]	20.0 [10.0]	19.6 [28.3]
NEU (10^9^/L)	3–12.5	2.4 [31.0]	10.0 [20.0]	10.9 [37.0]
LYM (10^9^/L)	1–4	4.8 [21.4]	10.0 [40.0]	8.7 [26.1]
MONO (10^9^/L)	0.1–1.35	33.3 [11.9]	50.0 [20.0]	30.4 [15.2]
EOS (10^9^/L)	0.1–1.25	95.2 [0.0]	90.0 [10.0]	95.7 [0.0]

ALB (g/L)	28–40	45.2 [0.0]	80.0 [0.0]	69.6 [0.0]

HCT: Haematocrit; RBC: red blood cells; HB: haemoglobin; MCV: mean corpuscular volume; MCH: mean corpuscular haemoglobin content; MCHC: mean corpuscular haemoglobin concentration; RDW: red cell distribution width; PLT: platelets; WBC: white blood cells; NEU: neutrophils; LYM: Lymphocytes; MONO: monocytes; EOS: eosinophils; ALB: albumin.

## Data Availability

The data used to support the findings of this study are available from the corresponding author upon request.
